# Supplementary material for: The Evolution and Application of a Novel DNA Aptamer Targeting Bone Morphogenetic Protein 2 for Bone Regeneration
Source: Molecules. 2024 Mar 11;29(6):1243. doi: 10.3390/molecules29061243 (PMC10974264; doi:10.3390/molecules29061243)
Supplement: Supplementary file 1 [file molecules-29-01243-s001.zip › molecules-2883235-supplementary.pdf]

Supplementary Informations

# Evolution and Application of a Novel DNA Aptamer Targeting Bone Morphogenetic Protein 2 for Bone Regeneration

**Mengping Liu [1], Andrew B. Kinghorn [1], Lin Wang [1], Soubhagya Bhuya [1], Simon Chi-Chin Shiu [1] and Julian A. Tanner [1,2*]**

[1] School of Biomedical Sciences, Li Ka Shing Faculty of Medicine, The University of Hong Kong, Pokfulam, Hong Kong SAR, People's Republic of China
[2] Advanced Biomedical Instrumentation Centre, Hong Kong Science Park, Shatin, New Territories, Hong Kong SAR, People's Republic of China

* Correspondence: jatanner@hku.hk (J.A.T.)

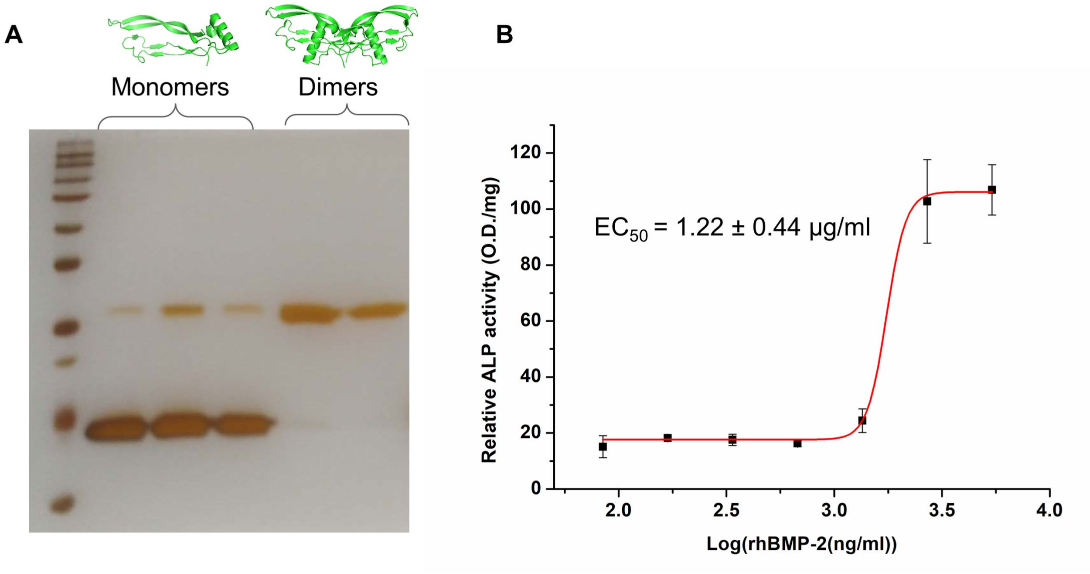


**Figure S1.** Expression and characterization of rhBMP-2 in *E. coli*. (A) Expression, refolding, and purification of rhBMP-2 in *E.coli*. Proteins after refolding were purified with heparin-affinity chromatography. Monomer and dimer fractions were analyzed with 12% SDS-PAGE gel electrophoresis and visualized with silver staining. (B) Determination of the ALP activity of *E.coli*-expressed rhBMP-2 dimers (n=3, three independent replicates ). The expression of ALP in C2C12 cells was induced by the treatment of rhBMP-2 for three continuous days. $EC_{50}$ was estimated under the non-linear fit model using the function of Dose Response in Origin 2021.

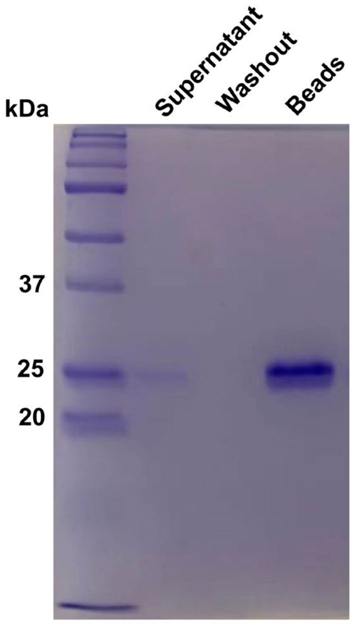


**Figure S2.** Saturation of nickel beads by rhBMP-2. RhBMP-2 was incubated with nickel beads for 1 hour and washed with the selection buffer three times. After the incubation, proteins in the supernatant, third wash, and beads were analyzed by 12% SDS-PAGE gel electrophoresis and stained with Coomassie blue. RhBMP-2: 20 μg; Nickel beads: 10 μl.

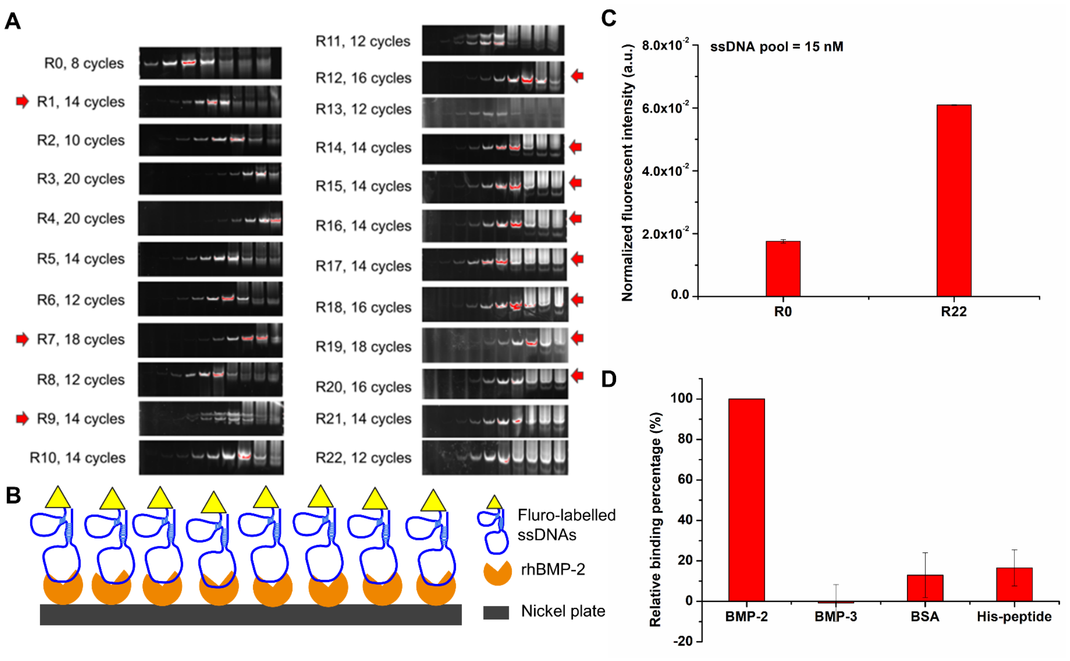


**Figure S3.** Aptamer selection cycle progress. (A) Evaluation of the enrichment progress vis PCR cycles. The place indicated by the red arrow means that the stringency was enhanced in this selection round. R0-22, BMP-2-binding ssDNA collected from the 0 to 22$^{nd}$ round of selections. The input of the ssDNA library was fixed in each selection, therefore fewer PCR cycles indicate more enrichment of the ssDNA pool. (B) Principle of the fluorescent plate assay. Fluor-labelled ssDNA was generated from PCR products of target ssDNA amplified with Alexa Fluor 488-labelled forward primers. (C) Affinity evaluation of R22 via the fluorescent assay. The nickel plate was saturated by rhBMP-2 before use. ssDNA: 15 nM. The fluorescent signal was measured by the microplate reader with excitation = 499 nm and emission = 520 nm. (D) Specificity evaluation of R22 via the fluorescent assay. ssDNA: 15 nM.

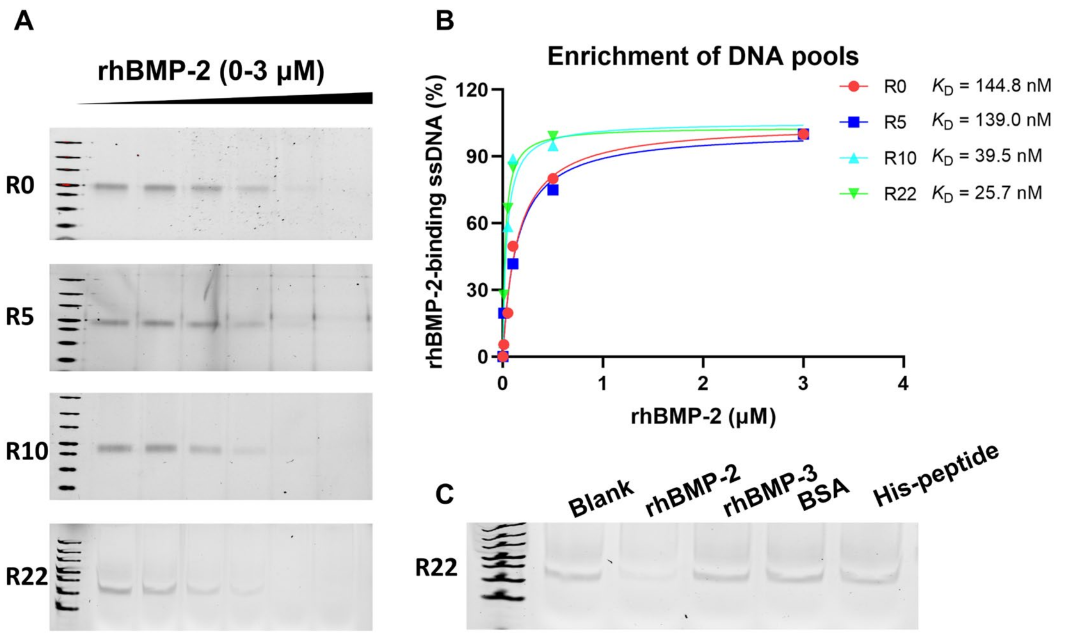


**Figure S4.** Enrichment progress of the ssDNA library against rhBMP-2. (A) Affinity evaluation of the ssDNA pool via EMSA. ssDNA samples were taken from R0, R5, R10, and R22. ssDNA pool: 6.5 nM; rhBMP-2: 0-3 μM. (B) Quantitative analysis of $K_D$ for each enriched library. EMSA data from panel A were quantified by Image J and $K_D$ values were computed by the Function Hyperbola in Graphpad Prism 8. (C) Specificity evaluation of R22 by EMSA. ssDNA pool: 6.5 nM; rhBMP-2: 0.1 μM.

A

| Group | Representative sequence | Cluster | Reads | RPM | Enrichment from R0 | Estimated ΔG (kcal/mol) |
|---|---|---|---|---|---|---|
| Non-adenine-rich group | BNA1 | - | 3 | 1.97 | 2 | -24.40 |
| | BNA2 | - | 4 | 2.63 | 2 | -23.30 |
| | BNA3 | - | 8 | 4.94 | 5 | -14.84 |
| Adenine-rich group | BA1 | 1 | 50,230 | 33,014 | 1135 | -6.39 |
| | BA2 | 2 | 95,809 | 62,971 | 1156 | -7.84 |
| | BA3 | 3 | 45,615 | 29,980 | 1211 | -7.47 |
| | BA4 | 4 | 33,818 | 22,227 | 691 | -6.79 |
| | BA5 | 5 | 33,589 | 22,077 | 1081 | -9.72 |

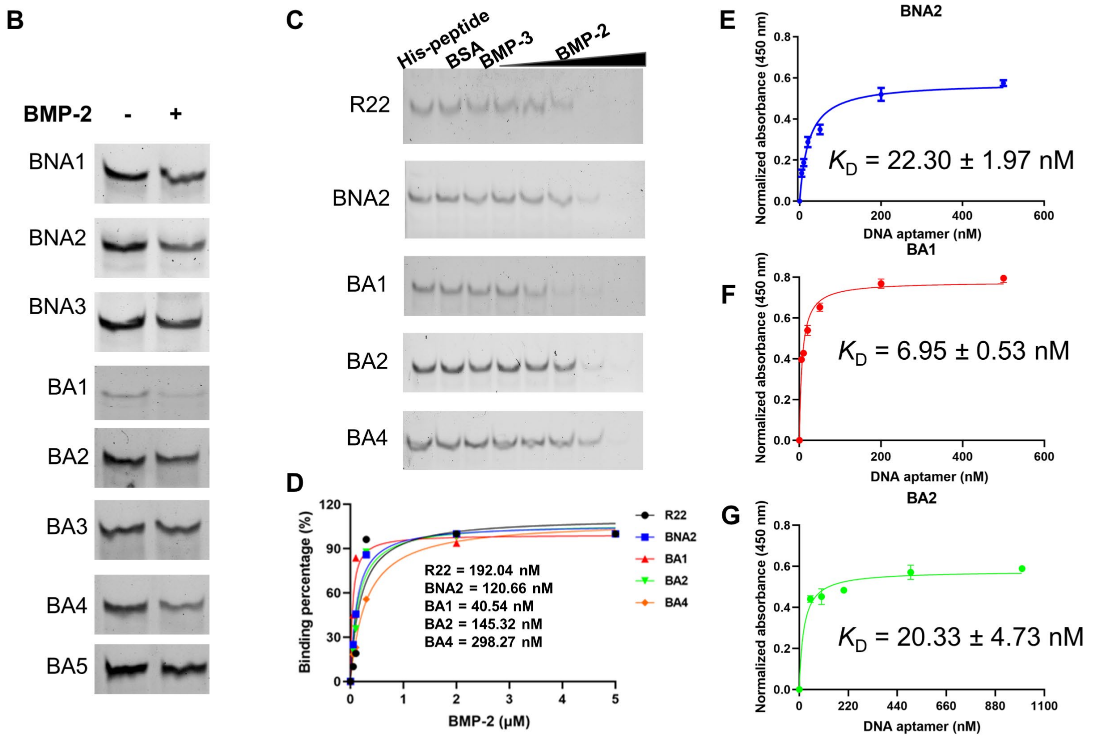


**Figure S5.** Affinity evaluation of rhBMP-2-binding aptamer candidates. (A) Selected aptamer candidates for characterization. BNA3 is from R10. BNA1-2 and BA1-5 were from R22. RPM: reads per million. Enrichment: the ratio of the copy number of the sequence to that in R0. Clusters were ranked in the order of their coverage in the enriched ssDNA pool. ΔG values were estimated with

Mfold. All sequences are presented in **Table 1**. (B) A quick screen of aptamer candidates to BMP-2 via EMSA. DNA: 25 nM; BMP-2: 1 μM. (C,D) Determination of $K_D$ values of several stronger aptamer candidates with EMSA. The intensities of ssDNA bands were measured with Image J and $K_D$ values were computed in panel C using the function Hyperbola under the non-linear fit model in GraphPad Prism 8. BMP-2: (0, 0.05, 0.3, 2, 5) μM. DNA: 25 nM. His-peptide/BSA/BMP-3: 0.3 μM. (E–G) Estimation of $K_D$ values of BNA2, BA1, and BA2 via ELONA (n=3, three independent replicates). BMP-2: 100 ng/well. DNA: 0-1000 nM. $K_D$ values were calculated in the same way as that in panel C.

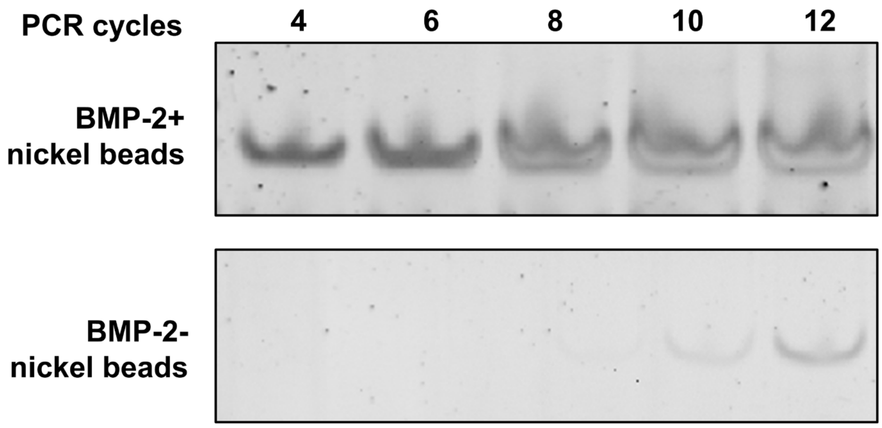


**Figure S6.** Evaluation of the binding of BA1 to nickel beads. 100 nM BA1 was incubated with BMP-2-immobilized or bare nickel beads for 30 min and selected BA1 was amplified with PCR for 4-12 cycles. The resultant PCR products were analyzed with 10% native PAGE gel.

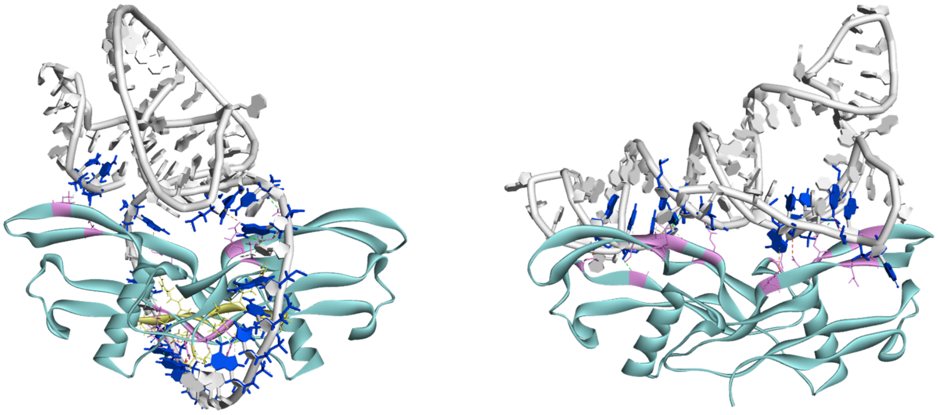

**BA1-BMP-2 interaction model**

**Scramble-BMP-2 interaction model**

| **Interaction model** | **Free energy (kcal/mol)** | **LGscore** | **MaxSub** | **Heparin-binding residue (%)** |
|---|---|---|---|---|
| BA1-BMP2 | -312.27 | 4.552 | 0.359 | 50% |
| Scramble-BMP2 | -255.46 | 4.552 | 0.359 | 0% |

**Figure S7.** Representation of interaction models of ssDNA-BMP-2 complexes. BA1 and scramble sequence are marked in grey and the BMP-2 structure is labelled in green. Interface residues in DNA sequences are marked in blue, and in BMP-2 are highlighted by yellow (heparin-binding site) and pink (non-heparin-binding region). The table shows the modelling outcome of two ssDNA-BMP-2 complexes. The heparin-binding residue (%) indicates the percentage of BMP-2 interface residues involved in the heparin-binding domain. LGscore and MaxSub are measured to determine the model quality which approximately reaches the good standard (3<Lgscore<5, 0.1<MaxSub<0.5).

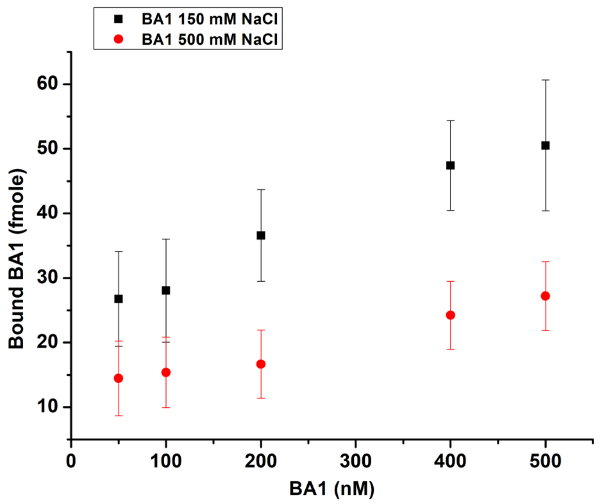


**Figure S8.** Evaluation of the binding affinity of BA1 under low and high salt conditions. Bound BA1 to BMP-2 were detected with ssDNA Qubit assay. BA1: 0-500 nM; BMP-2: 300 ng/well. BMP-2 was immobilized on nickel-coated black plate wells.

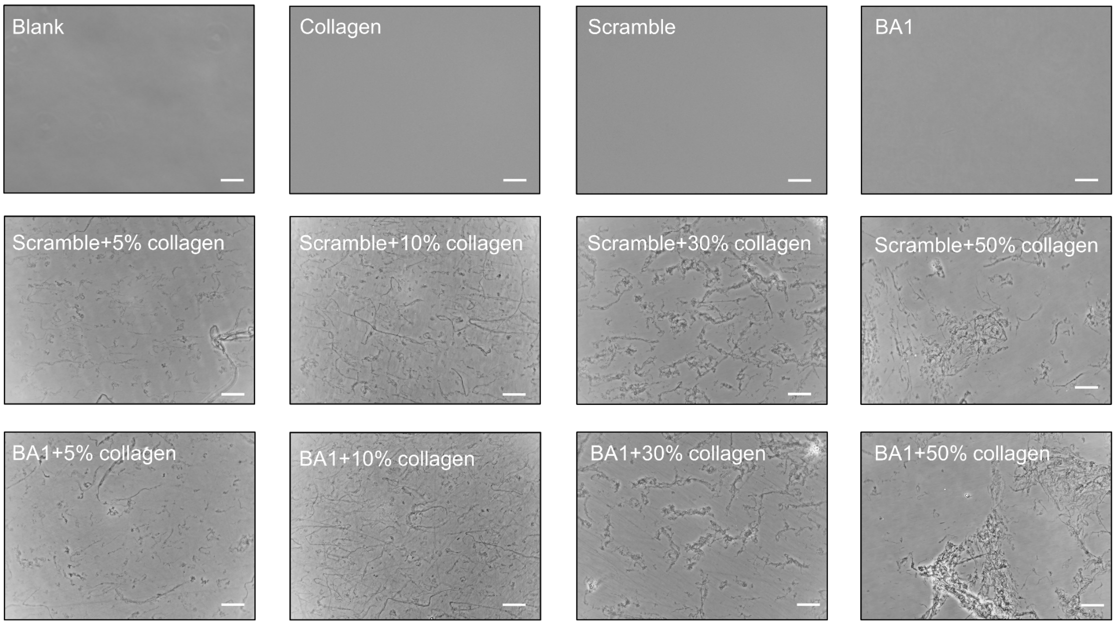


**Figure S9.** Morphology of DNA-collagen fibers. These DNA-collagen fibers were constructed with 500 nM DNA and 0.3 mg/ml collagen at different volume fractions (5%, 10%, 30%, and 50%). Photographs of these fibers were taken by the inverted microscope after an overnight assembly at room temperature. Scale bar: 40 μm

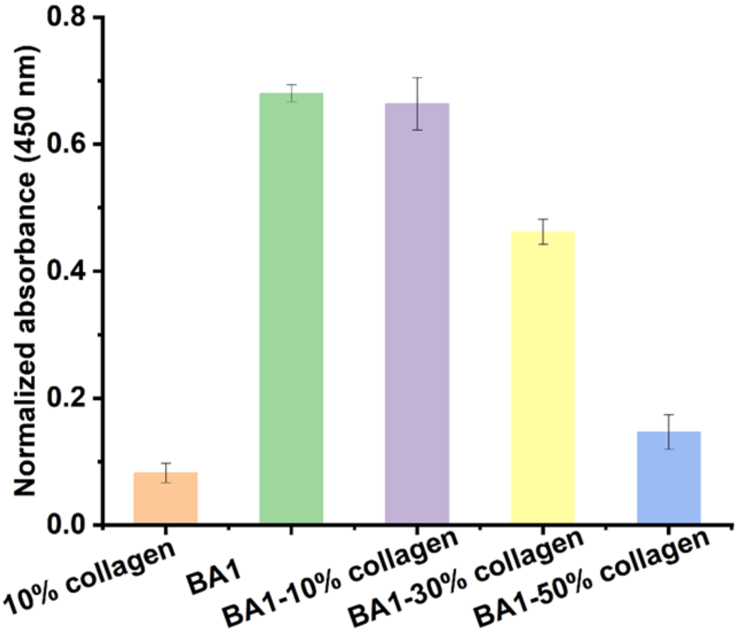


**Figure S10.** Study of the BMP-2-binding affinity of BA1 on different fibrous scaffolds (n=3, three independent replicates). Fibers were assembled with 500 nM BA1 and 0.3 mg/ml collagen at different volume fractions (10%, 30%, and 50%). BMP-2 was incubated with surface-immobilized fibers and determined with the ELISA-like assay. Absorbance signals were normalized and measured at 450 nm.

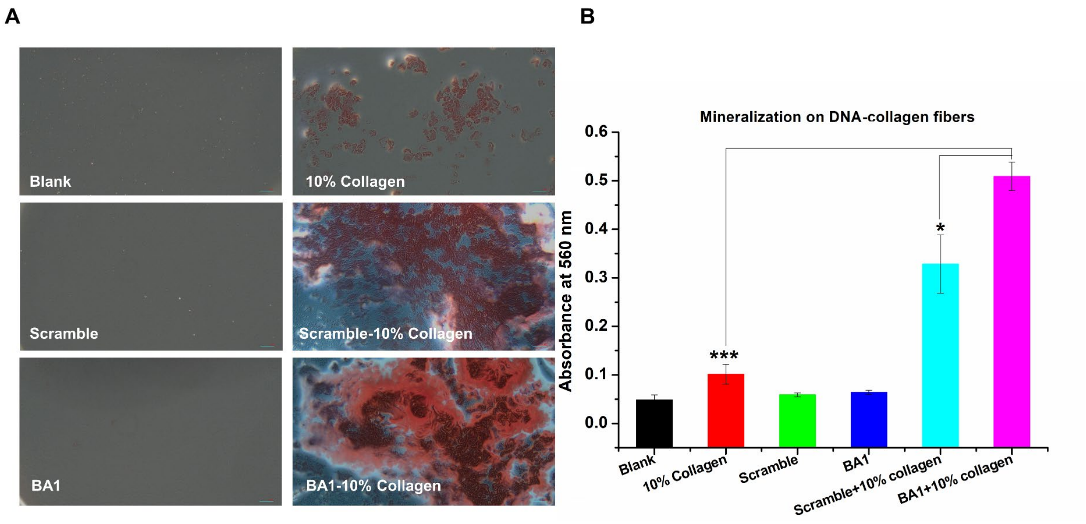


**Figure S11.** Characterization of calcium formed in DNA-collagen fibers (n=3, three independent replicates). (A) Microscopic images of calcium developed in different systems. DNA-collagen fibers were assembled with 500 nM DNA and 10% (v/v) 0.3 mg/ml type I collagen. Minerals were induced by the mineralization buffer containing 25 mM NaCl, 8 mM $Na_2HPO_4$ and 15 mM $CaCl_2$ for overnight incubation at room temperature. Calcium deposits were stained with alizarin red. (B) Quantitative analysis of calcium crystals formed in panel A. Calcium deposits stained with ARS were extracted by 10% (v/v) cetylpyridinium chloride (CPC) solution and the absorbance was measured at 560 nm. *P<0.05, ***P<0.001. BA1-10% collagen vs 10% collagen, P = $0.4706\times10^{-6}$; BA1-10% collagen vs Scramble-10% collagen, P = 0.0316.

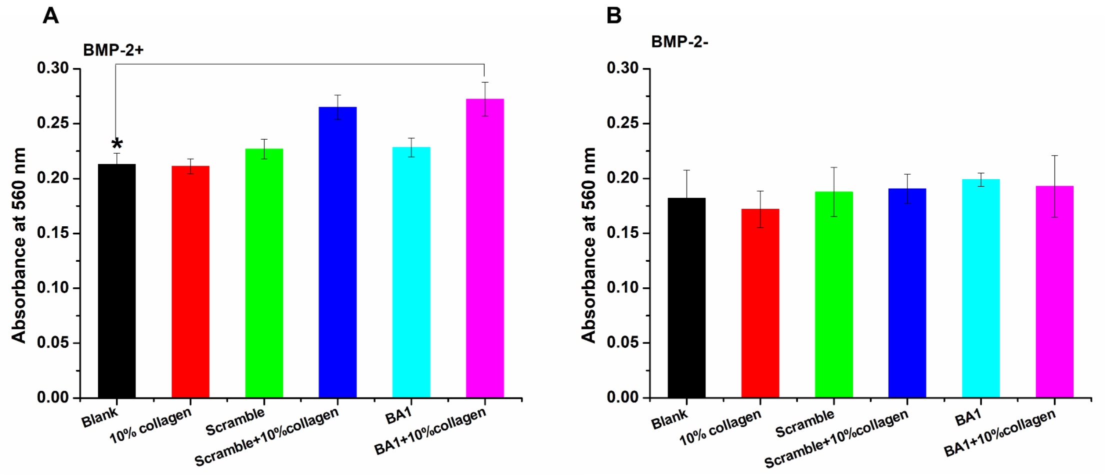


**Figure S12.** Determination of the formation of calcium in C2C12 cells induced by BMP-2 loaded on different scaffolds (n=3, three independent replicates). (A) and (B) quantified calcium in C2C12 cells with or without the treatment of free BMP-2 or BMP-2 loaded on different systems. DNA-collagen fibers were fabricated with 500 nM DNA and 10% (v/v) 0.3 mg/ml type I collagen. Calcium deposits were stained with ARS and further extracted by 10% CPC for the absorbance measurement at 560 nm. $^{*}P<0.05$, BA1-10% co
